# The Synergistic Effect of Leukocyte Platelet-Rich Fibrin and Micrometer/Nanometer Surface Texturing on Bone Healing around Immediately Placed Implants: An Experimental Study in Dogs

**DOI:** 10.1155/2016/9507342

**Published:** 2016-11-30

**Authors:** Rodrigo F. Neiva, Luiz Fernando Gil, Nick Tovar, Malvin N. Janal, Heloisa Fonseca Marao, Estevam Augusto Bonfante, Nelson Pinto, Paulo G. Coelho

**Affiliations:** ^1^Department of Periodontology, University of Florida, 1395 Center Drive, D1-11, Gainesville, FL 32610, USA; ^2^Department of Dentistry, Universidade Federal de Santa Catarina, R. Eng. Agronômico Andrei Cristian Ferreira, s/n Trindade, 88040-900 Florianópolis, SC, Brazil; ^3^Department of Biomaterials and Biomimetics, New York University, 433 1st Ave., Room 844, New York, NY 10010, USA; ^4^Department of Epidemiology and Health Promotion, New York University, 345 E 24th Street, New York, NY 10010, USA; ^5^Department of Prosthodontics and Periodontology, University of Sao Paulo, Bauru School of Dentistry, Alameda Octávio Pinheiro Brisolla 9-75, 17.012-901 Bauru, SP, Brazil; ^6^Department of Periodontics and Implant Dentistry, Faculty of Dentistry, University of the Andes (UANDES), Mons. Alvaro del Portillo 12.455, Las Condes, Santiago, Chile; ^7^Hansjörg Wyss Department of Plastic Surgery, NYU Langone Medical Center, 550 1st Avenue, New York, NY 10016, USA

## Abstract

*Aims*. This study evaluated the effects of L-PRF presence and implant surface texture on bone healing around immediately placed implants.* Methods*. The first mandibular molars of 8 beagle dogs were bilaterally extracted, and implants (Blossom™, Intra-Lock International, Boca Raton, FL) were placed in the mesial or distal extraction sockets in an interpolated fashion per animal. Two implant surfaces were distributed per sockets: (1) dual acid-etched (DAE, micrometer scale textured) and (2) micrometer/nanometer scale textured (Ossean™ surface). L-PRF (Intraspin system, Intra-Lock International) was placed in a split-mouth design to fill the macrogap between implant and socket walls on one side of the mandible. The contralateral side received implants without L-PRF. A mixed-model ANOVA (at *α* = 0.05) evaluated the effect of implant surface, presence of L-PRF, and socket position (mesial or distal), individually or in combination on bone area fraction occupancy (BAFO).* Results*. BAFO values were significantly higher for the Ossean relative to the DAE surface on the larger mesial socket. The presence of L-PRF resulted in higher BAFO. The Ossean surface and L-PRF presence resulted in significantly higher BAFO.* Conclusion.* L-PRF and the micro-/nanometer scale textured surface resulted in increased bone formation around immediately placed implants.

## 1. Introduction

The phenomenon of osseointegration has allowed multiple specialties in dentistry and medicine to better rehabilitate form and function of a multitude of clinical scenarios that include tissue loss due to pathology or trauma. In dentistry, one of the most commonly encountered clinical situations is progressive alveolar bone loss that occurs after tooth extraction. It has been well characterized that this progressive loss may substantially reduce the ridge dimensions prior to implant placement resulting in less favorable clinical conditions both functionally and aesthetically, before an implant is placed on the healed ridge [1–3].

In an attempt to improve the distribution and quantity of bone present around implants, along with decreasing treatment time for final prosthetic rehabilitation, minimally traumatic extraction techniques followed by immediate implant placement have been widely utilized and investigated [4–6]. Given the large number of preclinical and clinical investigations on this topic available in the literature, manuscripts have focused on currently utilized techniques and biomaterials associated, to systematically review survival outcomes and success of bone augmentation procedures, and most recently on esthetic outcomes [4–6].

Regardless of the surgical technique utilized, it is unequivocal that the extraction socket cervical dimensions are larger than the implant diameter and that a gap is going to be present between the implant and extraction socket walls [6]. Hence, a blood clot that forms between implant and socket walls and woven bone formation bridging the gap between implant allows for structural continuity between bone in intimate contact with surface and new bone formed due to socket healing [1–3, 7].

While this gap may be bridged with new bone forming within the extraction socket, several reports indicate that grafting procedures may be indicated to avoid soft tissue downgrowth between implant and the socket walls [8–11]. The graft material can be associated or not with a barrier membrane. This approach has been shown in both animal and clinical studies to increase the level of implant osseointegration, help maintain crestal bone levels, and improve esthetic outcomes, due to appropriate gap scaffolding that avoids soft tissue downgrowth at the gap region [12]. Despite improvements in clinical outcomes, graft material short- and long-term turnover rates, specially of xenografts and synthetic bioactive ceramics, are questionable due to the slow degradation characteristics of these materials, since slow graft material degradation and turnover may affect tissue quality and composition at the implant interface with the oral cavity [12]. Along with increased degrees of implant osseointegration reported in multiple manuscripts, a recent critical review by Wang and Lang [6] has pointed that concomitant guided bone regeneration (GBR) along with immediately placed implants may partially compensate alveolar bone resorption. Wang and Lang also pointed that GBR techniques utilizing various particulate materials were effective in ridge dimension preservation and that the application of GBR principles using bone substitutes along with a collagen membrane has shown clear effects on preserving alveolar ridge height and width [6].

Autologous tissue engineering approaches such as platelet-rich plasma (PRP), fibrin glues, and platelet-rich fibrin have been utilized in both orthopedic and oral and maxillofacial surgery in an attempt to hasten both bone and soft tissue healing [13, 14]. A recently published case report has presented promising results in socket and ridge maintenance after tooth extraction by utilizing a leucocyte and platelet-rich fibrin (L-PRF) along with an xenograft as a scaffolding biomaterial between immediate implant and fresh extraction socket wall [15]. Since L-PRF may be engineered to present easily shapeable resilient membrane forms that carry cells that may enhance both hard and soft tissue healing through the sustained release over time of growth factors from active platelets, leukocytes, and other circulating cells, such biomaterial may be presented as an alternative to classic GBR procedures for socket/ridge preservation with or without immediate implant placement [13]. Extensive reviews have elaborated on the potential use of L-PRF on periodontal [16] and reconstructive oral surgeries for dental implant treatment [17] with promising outcomes, reportedly due to its unique fibrin architecture and leukocyte content when compared to PRP [18]. In addition, depending on implant surface topographic characteristics in multiple length scales [19–21], higher degrees of interaction between surface and the high fibrin content of L-PRF may provide a seamless pathway for osteogenic cell migration between the healing socket walls and the implant surface, facilitating the device osseointegration [19–21]. The specific interaction between L-PRF and nanoscale topography within a larger-scale microtopography, fabricated by robotic microblasting of a resorbable blasting media powder, warrants investigation. Previous work has shown promising results for this nanoenabled surface with a substantial increase in osseointegration parameters compared to alumina-blasted acid-etched [22] and to dual acid-etched surfaces [23]. Thus, the aim of this investigation was to morphologically/metrically evaluate the effect of L-PRF and implant surface texture on bone healing around implants placed immediately after tooth extraction in a beagle dog model. The postulated hypothesis was that the combination of L-PRF with an implant surface presenting micrometer/nanometer scale texturing would result in higher degrees of osseointegration of immediately placed implants.

## 2. Materials and Methods

### 2.1. Study Design

#### 2.1.1. Implants

This study utilized screw root-form Blossom implants (Intra-Lock International, Boca Raton, FL) of 3.75 mm in diameter and 13 mm in length. Two different implant surfaces were utilized, namely, a dual acid-etched (DAE) surface and the commercially available Ossean surface (Intra-Lock International, Boca Raton, FL). Both surfaces have previously been characterized, where the DAE surface presents textured micrometer scale and smooth nanometer scale whereas the Ossean surface presents textured nanometer scale within the textured micrometer scale [24]. A total of 16 implants of each surface were utilized in the present study.

#### 2.1.2. Preclinical In Vivo Model

Following approval of the bioethics committee for animal experimentation at the Ecole Nationale Veterinaire D'Alfort, France, 8 beagle dogs (approximately 2-year-old dogs) were acquired for the study and allowed to acclimate for 2 weeks prior to surgery. All surgical procedures were performed under general anesthesia. The preanesthetic procedure comprised an intramuscular (IM) administration of acepromazine maleate (0.2 mg/kg), diazepam (0.5 mg/kg), and fentanil (4 mg/kg). Anesthetic induction was then achieved through ketamine (3 mg/kg), and general anesthesia was then obtained and maintained by 1 to 2% halothane.

**Figure 1:**
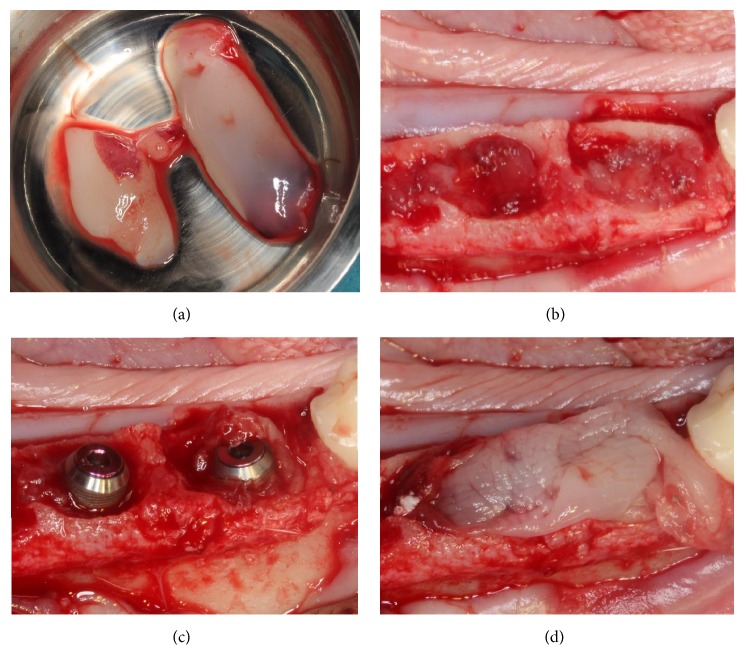
(a) L-PRF after processing and (b) its placement into the mesial and distal molar extraction sockets prior to (c) implant placement. (d) An extra L-PRF layer was placed over the implants prior to soft tissue closure.

**Figure 2:**
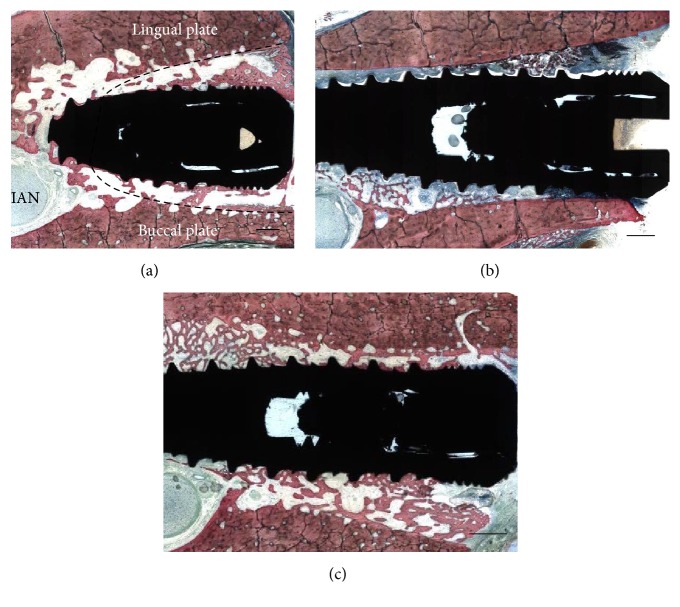
(a) Observation of histologic sections depicted the implant placed in the center of the socket (estimated by the dotted line) in proximity with the inferior alveolar nerve (IAN). The gap distance at the time of implant placement between the implant and buccal/lingual plates was also observed at six weeks and presented newly formed bone partially filling this gap. Bar = 2 mm. Representative histologic sections for implants placed in sockets (b) without L-PRF and (c) with L-PRF. The absence of PRF around the implant in (a) resulted in partial soft tissue apical migration in the gap comprised by the implant and extraction socket wall, while soft tissue apical migration in (b) was avoided by the presence of the L-PRF scaffold. No notable differences in socket healing pattern were observed between surfaces regardless of implant placement with or without L-PRF. Bar = 3 mm for (b and c).

Bilateral extractions of the mandibular first molars were performed. Mucoperiosteal flaps were elevated and teeth were sectioned in the buccolingual direction to allow nontraumatic individual root extraction by means of root elevators and forceps. One implant of each surface was placed in a split-mouth design at the mesial or distal extraction sockets in an interpolated fashion per animal so that the number of DAE and Ossean implant surfaces was equally distributed per mesial and distal sockets. On one side of the mandible, the implants were placed to the level of the buccal bone plate and soft tissue closure was achieved through standard suture procedures. On the contralateral side, L-PRF was prepared from each individual animal, by drawing venous blood using proprietary tubes and spinning them through a proprietary centrifuge at 2700 rpm for 12 minutes, or 400 RCF (CE/FDA cleared, IntraSpin, Intra-Lock, Boca Raton, FL) (Figure 1(a)). Both mesial and distal sockets were then filled with L-PRF (Figure 1(b)) and the implants placed within the socket to the buccal plate level. L-PRF in a membrane shape, obtained from the XPression fabrication kit, (Intra-Lock, Boca Raton, FL) was placed over the implants prior to standard soft tissue suture closure (Figure 1(d)). After placement, a minimum gap of 2 mm was left between the implant and the buccal plate (Figure 1(c)). Drilling direction aimed to avoid invasion of the lingual plate during osteotomy preparation or after implant placement (Figure 2). Remarkably similar implant placement patterns were observed due to the split-mouth design where one side did receive L-PRF whereas the other did not. Healing cover screws were adapted to the implant internal connection. Flaps were repositioned and sutured on both sides of the mandible with 4.0 PGA suturing material (Vicryl, Ethicon Johnson & Johnson, Miami, FL, USA). Postsurgical medication included IM administration of antibiotics (Cefazolin 30 mg/kg every 12 hours for 3 days) and anti-inflammatory (0.2 mg/kg per day for 3 days). Euthanasia was performed by anesthesia overdose, 6 weeks after implant placement.

#### 2.1.3. Histological Preparation and Histomorphometry

At necropsy, the mandibles were retrieved by sharp dissection. The implants in bone were then separated from the mandible allowing blocks containing the implants in the mesial and distal sockets. The bone blocks were kept in 10% buffered formalin solution for 24 h and gradually dehydrated in a series of alcohol solutions ranging from 70 to 100% ethanol. Following dehydration, the samples were embedded in a methacrylate-based resin (Technovit 9100, Kulzer & Co, Wehrheim, Germany) according to the manufacturer's instructions. The sections, performed in a buccal-lingual direction, were then reduced to a final thickness of ~30 *μ*m by means of a series of diamond blade sectioning and SiC abrasive papers (400, 600, 800, 1200, and 2400 Grit) in a grinding/polishing machine (Metaserv 3000, Buehler, Lake Bluff, IL, USA) under water irrigation [25]. The sections were then subjected to the Stevenel's Blue and Van Gieson staining technique. The percentage of bone area fraction occupancy (BAFO) was determined at a 50x magnification (Leica DM4000, Wetzlar, Germany) with the aid of computer software (Image J, NIH, MD, USA).

Statistical analysis was performed by mixed model ANOVA (at *α* = 0.05). The statistical unit utilized for evaluation was the number of animals. The independent variables considered (individually or in combination) were implant surface, socket position (mesial or distal), and the presence of L-PRF. The dependent variable considered was BAFO.

## 3. Results

With the exception of one animal that developed a localized infection two weeks after surgery (excluded from statistical analysis), no complications were observed during animal surgical procedures or follow-up assessments, including postoperative infection or any other clinical concern.

**Figure 3:**
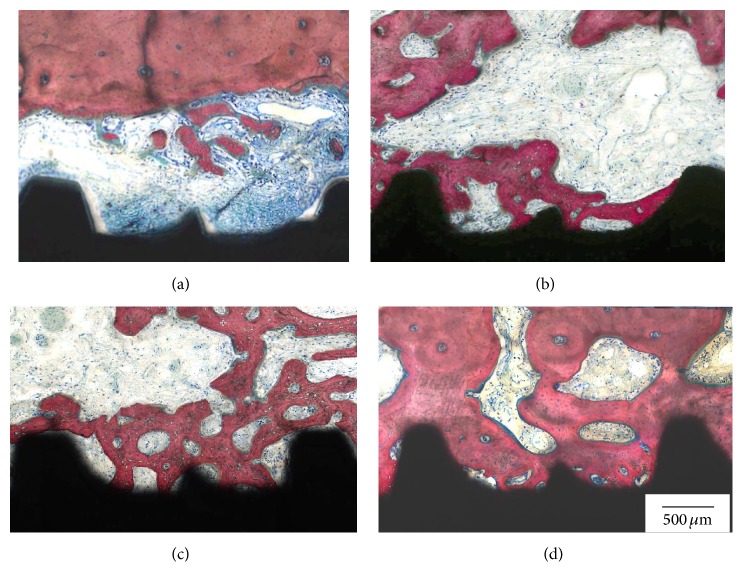
Representative histologic sections of the bone/implant interface for the (a) DAE without L-PRF, (b) Ossean without L-PRF, (c) DAE with L-PRF, and (d) Ossean with L-PRF.

General histologic analysis depicted the implant placed in the center of the socket at a distance from the buccal plate in proximity with the inferior alveolar nerve (Figure 2(a)). The gap distance present at the time of implant placement between the implant and buccal/lingual plates was easily depicted at six weeks and presented newly formed bone partially filling this gap. Representative histologic sections for implants placed in sockets with and without L-PRF are presented in Figures 2(b) and 2(c). The absence of L-PRF around implants most often resulted in partial soft tissue apical migration in the gap comprised by the implant and extraction socket walls, while soft tissue apical migration was avoided by the presence of the L-PRF scaffold between implant and socket walls. No notable differences in socket healing pattern, comprised by an intramembranous-like ossification pattern occurring between implant and socket walls, were observed between surfaces regardless of implant placement with or without L-PRF. Contact osteogenesis was observed for both groups through direct bone apposition onto both implant surfaces, and qualitatively higher amounts of bone were observed in the proximity with both implant surfaces placed in sockets filled with L-PRF relative to implants placed into sockets filled with blood clot (Figure 3).

**Figure 4:**
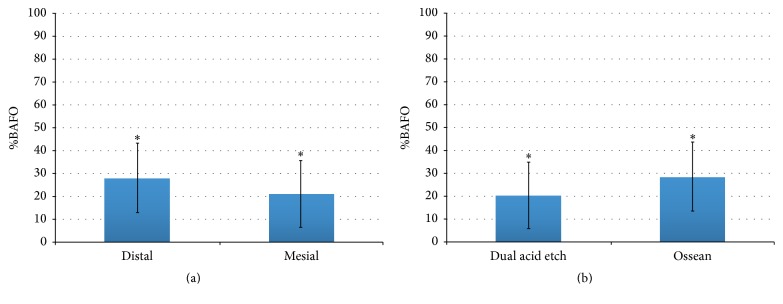
Statistical summary of (a) BAFO when surface and L-PRF presence were collapsed over socket position showed no statistical differences. Statistical summary of (b) BAFO when socket position and L-PRF presence were collapsed over implant surface showed higher mean values for the Ossean surface relative to the DAE surface. However, no statistical differences were detected. The same number of asterisks depicts statistically homogeneous groups.

When BAFO values were collapsed over implant surface and the presence or not of L-PRF and evaluated as a function of socket position, no significant differences in BAFO (*p* = 0.47) were observed between groups despite lower mean values for dependent variables observed for the mesial socket relative to the distal socket (Figure 4(a)). When BAFO was evaluated as a function of implant surface (collapsed over socket position and the presence or not of L-PRF), no significant differences in BAFO (*p* = 0.11) were observed despite higher mean values for both dependent variables observed for the Ossean surface relative to the DAE surface (Figure 4(b)).

**Figure 5:**
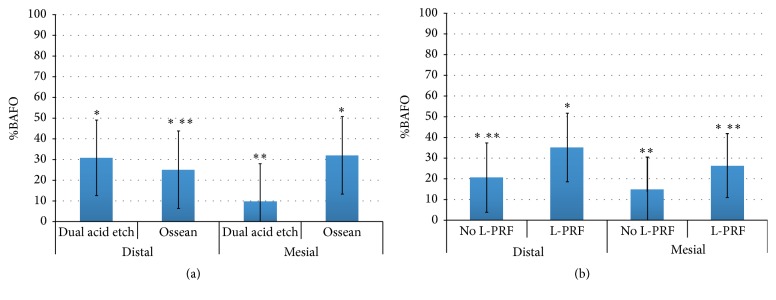
Statistical summary of (a) BAFO when L-PRF presence was collapsed over implant surface and PRF presence. While no effect of implant surface was detected for BAFO when the distal socket was considered, the Ossean surface presented significantly higher BAFO than the DAE surface in the medial socket. Statistical summary of (b) BAFO when implant surface was collapsed over implant surface and socket position. While no effect of L-PRF presence was detected for each individual socket, higher mean values of BAFO were observed for the sockets presenting L-PRF. A significant difference was observed between the medial socket without PRF and the distal socket with L-PRF. The same number of asterisks depicts statistically homogeneous groups.

**Figure 6:**
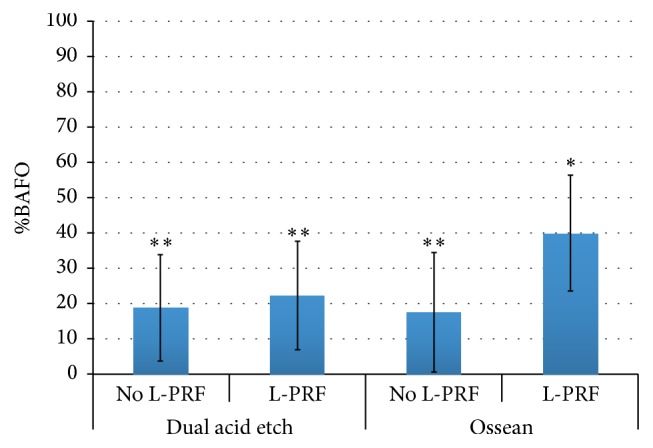
Statistical summary of BAFO when collapsed over socket position. A significant difference was observed when the Ossean surface was utilized with L-PRF relative to its counterpart without L-PRF. The same number of asterisks depicts statistically homogeneous groups.

While no effect of implant surface was detected for BAFO when the distal socket was considered, the Ossean surface presented significantly higher BAFO (*p* = 0.015) than the DAE surface in the mesial socket. A significant difference was observed between the mesial socket without L-PRF and the distal socket with L-PRF (*p* = 0.02). Finally, the combination of the Ossean surface and L-PRF presence resulted in significantly higher BAFO level relative to its no L-PRF counterparts and DAE surfaces with and without L-PRF (*p* = 0.012) (Figures 5 and 6).

## 4. Discussion

Dental implantology has been extensively researched in basic and clinical grounds and has for decades enjoyed the status of standard of care for the treatment of both partial and full edentulism. While reported high success rates [26, 27] have often been achieved through the classic protocol of delayed implant placement, when implants are placed at least twelve weeks following tooth extraction and following placement enough time is allowed for implant osseointegration and subsequent restoration, this classic approach may not necessarily lead to optimal treatment outcomes as other associated procedures may be required to further enhance clinical and esthetic outcomes [28]. Thus, modern clinical approaches that attempt to minimize tissue alteration after tooth extraction, while allowing for implant osseointegration in appropriate restorative positions for adequate function and esthetics, have been introduced and are currently under development [4–6].

A number of literature reviews on immediate implantation in fresh extraction sockets have evaluated the current state of the techniques [4, 6], biomaterials utilized [12], success, and esthetic outcomes [29] of these procedures. While it is general consensus that this treatment modality can be successful if well indicated, current materials utilized in an attempt to increase the degree of implant osseointegration by filling the gap between the implant and the socket walls include particulate grafting materials and barrier membranes. Despite improvements in clinical outcomes, slow graft material degradation and turnover may affect tissue quality and composition at the implant interface with the oral cavity [4, 6, 12].

The present investigation evaluated the effects of utilizing a validated tissue engineering approach through autogenous processed L-PRF [13] in an attempt to maximize osseointegration of implants placed in fresh extraction sockets. The rationale for testing the effect of L-PRF in this clinical situation was based on the natural origin of autogenous processed L-PRF and its potential to promote osteogenesis within the socket due to its dense fibrin mesh and cellular content that could potentially provide a sustained release of growth factors, as well as its potential to act as a physical barrier to avoid soft tissue downgrowth [13, 15].

The rationale for investigating two distinct types of implant surface was based on the basic phenomenon of osseointegration in fresh extraction sockets where large gaps between implant and socket walls are often encountered and have to be bridged for optimal biomechanical competence of the device [7, 30]. From an ideal perspective, the blood clot filling the space between implant and socket wall would bridge them subsequently leading to a seamless osteogenic tissue link between the device and socket walls where osteogenic cells may migrate in an intramembranous-like healing pathway that has been reported to occur from the periphery of the socket towards its center [6]. This ideal scenario may be disrupted by a variety of reasons that include soft tissue migrating and disrupting the fibrin bridge as well as blood clot contracting usually away from the implant surface towards the socket and/or osteotomy wall leaving an interrupted pathway for cell migration towards the implant surface [6]. Through the utilization of a DAE surface that presents micrometer scale texture and the Ossean surface that presents micrometer and nanometer scales texture [31], the study design provided the opportunity to test the hypothesis that the implant surfaces could influence osseointegration in the natural healing scenario (blood clot filled extraction sockets) and when a mechanically robust tissue engineered scaffold interposed the implant and socket walls facilitating the establishment of a seamless pathway between implant surface and socket wall. The study design employing a split-mouth arrangement between the contralateral presence or absence of L-PRF for implant surfaces placed in the same contralateral socket (distal or mesial depending on animal sequence) allowed direct comparison between groups that were nested within the same animal subject.

Given the multiple variables evaluated in the present study, general statistical analyses showed various trends when two independent variables were collapsed over remaining independent variables. In general, higher mean BAFO values were observed for the Ossean surface relative to the DAE surface, higher mean BAFO values were observed when L-PRF was utilized, and lower mean BAFO values were observed for the mesial socket relative to the distal socket (possibly explained by the larger size of the mesial socket relative to the distal socket that would result in larger gaps between implant and socket walls).

In general, the histomorphologic results obtained from implants placed in fresh extraction sockets are in direct agreement with previous studies [6, 7, 30, 32], where bone growth from the socket walls partially filled the gap between implant and the socket walls. Also in line with previous reports [6, 7, 30, 32], intimate contact between bone and implant surface was observed regardless of implant surface group, and partial apical migration of soft tissue occurred mainly through the gap present between implant and the socket buccal plate. Where no L-PRF was present, histometric BAFO were slightly favored by the presence of the Ossean surface. However, higher contribution of the Ossean surface was observed for BAFO (significant) when the analysis was restricted to the larger mesial socket, suggesting a surface effect when more challenging clinical scenarios are concerned.

When the presence or absence of L-PRF was evaluated as a function of socket position, substantially higher degrees of BAFO were observed for sockets filled with L-PRF. The substantial increase in BAFO observed for both sockets due to the presence of L-PRF is likely accounted by improved cell migration through the stable L-PRF scaffold present between implant and socket walls.

Our histometric results for BAFO further highlight the biologic and scaffolding properties of L-PRF when placed in combination with the Ossean implant surface. This result unequivocally demonstrates the synergistic effect that exists between a micrometer and nanometer length scale and the L-PRF structure that allows significantly higher amounts (~90%) of bone formation between implant threads than the combination of the DAE surface and L-PRF. A potential physical explanation for the BAFO discrepancy observed between DAE and Ossean surfaces placed with L-PRF lies in their roughness pattern differences [31], where less developed surface area is available for the micrometer scale textured DAE relative to the micrometer and nanometer scale textured Ossean surface. Such large difference in developed surface area between surfaces may have resulted in less efficient interaction between the DAE surface and the L-PRF scaffold that potentially resulted in discontinuities between the socket wall and implant surface, thus restricting seamless cell migration. Another possible reason for the significant BAFO differences between DAE and Ossean surfaces placed with L-PRF is that identical levels of interaction occurred between both surfaces and L-PRF and a pathway between the healing socket walls and implant was present. In this case, osteogenic cells would equally populate regions in close proximity with the implant surface but the highly osteogenic characteristics of the Ossean surface relative to the DAE surface [22, 31, 33] accounted for phenotype alteration and higher degrees of bone formation as previously presented in a comprehensive histomorphologic, histomorphometric, nanobiomechanical, and gene expression assessment study [31].

General histomorphologic observations for the implants placed along with L-PRF in fresh extraction sockets differed from those where no L-PRF was utilized primarily due to the lack of soft tissue migration through the gap formed between implant and socket wall, suggesting L-PRF's efficiency as a barrier during healing. The osteogenic potential and adequacy of L-PRF substituting the blood clot during socket healing were confirmed since bone growth occurred from the socket walls towards the implant leading to substantial bone formation around implants irrespective of implant surface group.

It should be acknowledged that this study is an in vivo preclinical model on beagle dogs, suggesting that the synergistic effect of leukocyte platelet-rich fibrin (L-PRF) and micrometer/nanometer surface texturing on bone healing around immediately placed implants should be evaluated in well-designed clinical trials in humans. Further studies are therefore needed to confirm the evidence emerging from the present research.

## 5. Conclusions

The postulated hypothesis that the combination of L-PRF with an implant surface presenting micrometer/nanometer scale texturing would result in higher degrees of osseointegration of immediately placed implants was accepted. Further studies are warranted to shed light on possible physical and molecular mechanisms that result in the substantial increase in the amount of bone in proximity with Ossean surface implant placed along with L-PRF.
